# Area-Selective Growth of Zinc Oxide Nanowire Arrays for Piezoelectric Energy Harvesting

**DOI:** 10.3390/mi15020261

**Published:** 2024-02-10

**Authors:** Frank Eric Boye Anang, Xuanwei Wei, Jiushuai Xu, Markys Cain, Zhi Li, Uwe Brand, Erwin Peiner

**Affiliations:** 1 Institute of Semiconductor Technology, TU Braunschweig, 38104 Braunschweig, Germany; x.wei@tu-braunschweig.de (X.W.); jiushuai.xu@tu-braunschweig.de (J.X.); e.peiner@tu-braunschweig.de (E.P.); 2 Scientific Metrology Department, Ghana Standards Authority, Accra P.O. Box MB 245, Ghana; 3 Electrosciences Ltd., Farnham, Surrey GU9 9QT, UK; markys.cain@electrosciences.co.uk; 4 Surface Metrology Department, Physikalisch-Technische Bundesanstalt (PTB), 38116 Braunschweig, Germany; zhi.li@ptb.de (Z.L.); uwe.brand@ptb.de (U.B.)

**Keywords:** area-selective growth, ZnO nanowire arrays, MEMS, CBD growth, SU-8 polymer, PENG

## Abstract

In this work, we present the area-selective growth of zinc oxide nanowire (NW) arrays on patterned surfaces of a silicon (Si) substrate for a piezoelectric nanogenerator (PENG). ZnO NW arrays were selectively grown on patterned surfaces of a Si substrate using a devised microelectromechanical system (MEMS)-compatible chemical bath deposition (CBD) method. The fabricated devices measured a maximum peak output voltage of ~7.9 mV when a mass of 91.5 g was repeatedly manually placed on them. Finite element modeling (FEM) of a single NW using COMSOL Multiphysics at an applied axial force of 0.9 nN, which corresponded to the experimental condition, resulted in a voltage potential of −6.5 mV. The process repeated with the same pattern design using a layer of SU-8 polymer on the NWs yielded a much higher maximum peak output voltage of ~21.6 mV and a corresponding peak power density of 0.22 µW/cm^3^, independent of the size of the NW array. The mean values of the measured output voltage and FEM showed good agreement and a nearly linear dependence on the applied force on a 3 × 3 µm^2^ NW array area in the range of 20 to 90 nN.

## 1. Introduction

Piezoelectric energy harvesting is a renewable-energy-harvesting technology that refers to the capturing of energy from mechanical vibration and converting it into electrical energy to power nano- and micro-scale devices that do not have access to an external power supply [1]. Here, piezoelectricity is used as the natural property of certain materials to generate electric potential when they are subjected to mechanical stimuli [2,3,4,5]. Therefore, it has the potential to extend the working life of many low-power (µW to mW) electronic devices. The concept of wireless systems relying on low-power sources with a long battery life has led to the development of microelectromechanical system (MEMS)-based vibrational or piezoelectric energy harvesters, otherwise known as piezoelectric nanogenerators (PENGs). Using the combination of piezoelectric and semiconducting properties, several external forces, like tiny vibrations and the bending of muscle, can be used to generate piezoelectric potentials [6]. The technology is used to solve the challenges associated with the use of traditional batteries, which include the limitation in the size of the device and the need for frequent recharging [7].

Nonetheless, there have been reported challenges with the realization of high-performance piezoelectric nanogenerators in ZnO due to the screening of the piezopotential generated under mechanical deformation by free carriers (electrons) [8,9,10]. The resulting low output voltage limits the electrical power that can be harvested with the device. A ZnO *p*-*n* homojunction with a lithium dopant that compensates for donor-free carriers and acts as a *p*-type polymer [8] has been reported to reduce the piezoelectric potential screening effect but cannot solve the problem when there is an inhomogeneous distribution of applied force on a nanowire (NW) array. In this case, the free charges from unstrained NWs drift toward the piezoelectric charges generated at the strained NWs, which reduces the output performance of the piezoelectric device. To overcome this challenge, it is important to reduce the surface free charge carriers in ZnO NWs. A further promising technology to reduce this screening effect is the separation of the total NW array into sub-patterns that are insulated from each other, e.g., using area-selective growth [8,10].

A PENG can be configured using two different designs, i.e., as a laterally integrated nanogenerator (LING) or a vertically integrated nanogenerator (VING) [3,11,12]. For the LING, piezoelectric NWs are first transferred from a growth substrate onto a receiver substrate. Metallic contact pads are then formed at the NW ends [12,13]. The VING is made up of arrays of vertically aligned NWs, which are grown on either a flexible or a rigid substrate, and is usually encapsulated in a dielectric polymer matrix with both top and bottom electrodes as contact pads. The VING configuration was shown to have higher output performance [14] and an easier fabrication process [2,11,12] compared with a LING. The induced piezopotential is strongly coupled to an external load/force in the VINGs by the conductive contact pads, which are normally deposited on a composite structure [15]. Depending on the type of substrate material and contact design in the LING/VING configuration used, a PENG can be operated under bending or compressive loading. The VING configuration was considered for compressive force application in our work.

Over the past two decades, ZnO has been investigated and shown to be a reliable material for piezoelectric energy harvesting owing to its coupled effect of piezoelectric and semiconducting properties [16,17]. It has a direct band gap of 3.4 eV and a large exciton binding energy of 60 meV, which makes it a material of interest in electronic applications, among others. Furthermore, it is a non-toxic, biocompatible material and has a high-electrochemical stability and a wide range of resistivity control (10^−3^ to 10^5^ Ω × cm) [7,18]. Compared with their bulk counterparts and thin films, ZnO NWs have a higher piezoelectric coefficient and larger elastic deformation when tiny physical stimuli are applied [2,9]. These attributes make ZnO NWs useful for micro/nanosensors and piezoelectric energy harvesting applications.

For sensing and energy-harvesting purposes, ZnO NWs have been synthesized via various physical and chemical methods, such as vapor-phase synthesis, metalorganic chemical vapor deposition (MOCVD), chemical bath deposition (CBD) [7,18,19,20,21], thermo-convective solution growth [22], and microwave synthesis [23]. Among these methods, the CBD technique has recently been embraced as one of the most effective, efficient, and high-performance growth methods for the fabrication of different nanomaterials and nanostructures. This is due to its advantages, such as low cost [19,24], reproducibility, cheap and readily available starting chemicals, low process temperature (<100 °C) [24], and use of environmentally friendly chemicals. As a low-temperature growth technique, the CBD method enhances well-aligned and well-controlled ZnO NW growth [25,26]. It also has the advantage of forming high-density arrays and high-quality crystals [18,27].

The expectation of higher output yield in PENGs, which can be hindered by the potential presence of intrinsic free carriers in ZnO upon mechanical deformation (also known as screening effect), is significantly reduced by the area-selective growth mode [8,10]. This is due to the possibility of the independent working of each patterned region. However, in order to effectively suppress the screening effect of free charge carriers, it is important to have the patterning done in much smaller sizes (µm^2^). In this case, a remarkable number of NWs is located at the edges of the arrays, where the carrier tunneling is suppressed and potential screening is much lower than those for NWs in the center [10]. In this paper, the area-selective growth process for uniformly aligned ZnO NW arrays (NWAs) on a cost-effective, easily synthesized (Zn sputtering and annealing), and compatible Si substrate using a low-temperature (<100 °C) aqueous solution growth method is reported as a proof of concept. This approach of area-selective growth using the CBD technique, which was preceded by Zn sputtering followed by oxidation to form ZnO, is unique and completely different from the ones reported in the literature. This process has the tendency to control the density, position, and alignment of ZnO NWAs on MEMS cantilever structures and over large areas of different sizes [20,21]. Separated NWAs have the capability of an elevated output of the nanogenerator device when the patterns are connected in series and in parallel for an increased voltage and current, respectively [8,28]. During the CBD-based two-step ZnO NW growth process, the ZnO seed layer (SL) is intended to serve three purposes, i.e., as a buffer layer to reduce the lattice mismatch between ZnO NWs and the silicon substrate, to assist the nucleation of ZnO on the mirror-like Si surface, and to facilitate the aligned growth of ZnO NWs perpendicular to the substrate surface [29]. In the present study, SL fabrication was achieved by direct-current (DC) sputtering of a Zn precursor layer, which was subsequently annealed in ambient air to oxidize it to ZnO [21]. An advantage of this procedure is that the ZnO SL can be patterned by lift-off of the Zn precursor layer using a photoresist. Thus, it can be easily integrated into a MEMS fabrication process, e.g., of a piezoresistive cantilever spring–mass resonator [20]. This patterning option has not been reported so far with sputtered [8,10,19,30], dip-coated [13,22], or spin-coated [31,32] ZnO SLs.

The performance of the proposed PENG on patterned surfaces of Si substrates was tested with two PENG designs: first, an NG device was fabricated without additional material on top or embedded between the NWs; the second design involved an SU-8 polymer matrix deposited on the NW array (NWA). Furthermore, the choice of polymer for a particular application in MEMS depends on many factors, prominent being the material properties, processing conditions, and performance demands of the device under consideration. They may be utilized as structural or functional components, as well as flexible substrates to encompass other devices [33]. Several polymer materials, including SU-8, polydimethylsiloxane (PDMS) [12,13], and polymethyl methacrylate (PMMA) [26,34], have been investigated for MEMS application. Amongst these, we settled on SU-8 due to its wide use as a free-film substrate and structural component for other devices. Other advantages include the compatibility of SU-8 with standard micro-machining processes, like photolithography and wet or dry etching. Multiple exposure steps on multiple layers that can be released in a single development are also possible with SU-8 [33]. Details of the experimental procedure are outlined in the following section.

## 2. Materials and Methods

**Figure 1:**
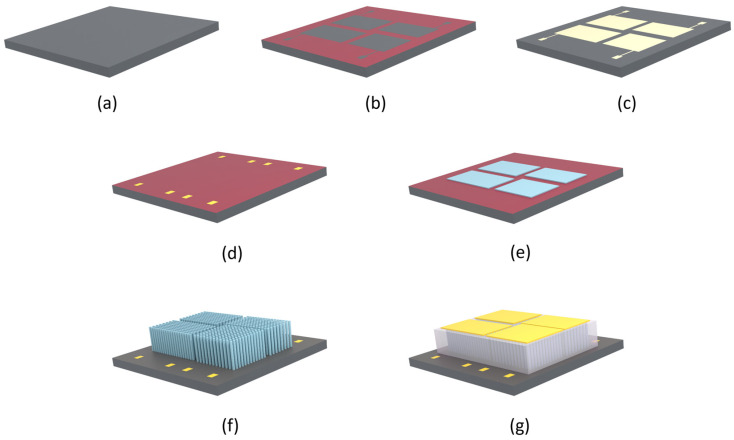
Fabrication steps of patterned-growth ZnO NW arrays (NWAs) on Si substrate for a PENG: (**a**) Si substrate; (**b**) photolithography and removal of SiO_2_ layer; (**c**) phosphorus doping diffusion for bottom contact; (**d**) photolithography, bottom, and top Cr/Au contact pads; (**e**) photolithography and Zn seed layer sputter coating; (**f**) CBD growth of ZnO NW arrays; (**g**) NWs encapsulation in polymer matrix with top Cr/Au contact pads.

The fabrication steps are illustrated in Figure 1. The process started with the dicing of a silicon substrate into four smaller pieces of size 30 × 30 mm^2^. The fabrication processes and parameters were the same for each sample. The Si wafer with <100> crystal orientation, 1–10 Ω × cm resistivity, thickness of 275 ± 15 µm, and a diameter of 100 ± 0.13 mm was purchased from Si-Mat Silicon Materials, Kaufering, Germany (Figure 1a). The substrates were dipped in a hydrofluoric (HF) acid mixture consisting of 30% H_2_O_2_ and 96% H_2_SO_4_ in a 1:1 volume ratio for 5 min to remove the native oxide layer on the surface. They were further rinsed in deionized water to remove the residual HF solution and then dried with nitrogen gas. The samples were then placed in a high-temperature furnace at 1100 °C to yield a silicon oxide (SiO_2_) layer (~300 nm thick) to be used as a mask in a later diffusion process (Figure 1b).

In the initial stages of patterning of the oxide layer, photolithography was performed. The samples were spin-coated with a positive photoresist (AZ 5214E, purchased from Micro Resist Technology, Berlin, Germany) at a speed of 5000 rpm for 30 s forming a homogeneous photoresist layer and subsequently soft-baked at 110 °C for 50 s. The formation of patterned regions was performed using the mask and an MJB4 mask aligner (SUSS MicroTec AG, Garching, Germany). The patterned samples were then immersed in a developer solution (AZ 726) for about 50 s. The oxide layer in the patterned areas was etched by placing the samples in an aqueous hydrofluoric acid solution (HF, 6–7%) for 10 min. The samples were cleaned using acetone in an ultrasonic bath to remove the photoresist. For the phosphorus doping, a phosphorus-containing emulsion (P509 Spin-on dopant from Filmtronics Inc., Butler, PA, USA) was spin-coated on the Si substrates and placed in a high-temperature furnace at 1100 °C for 30 min (Figure 1c). This allowed the phosphorus atoms to diffuse into the silicon and create a highly conductive *n*-type layer at its surface. Through the lithography-controlled process, a defined conductive circuit area was created on the Si surface for each pattern of the NG device. The phosphorus-doping process was necessary since the conductive layer at the Si surface was to be used as the bottom electrode for the final device instead of a metal layer. After a second oxidation step (Figure 1d), lithography and patterning by etching in aqueous hydrofluoric acid solution (HF, 6–7%) and electron-beam evaporation of Cr/Au (30 nm/300 nm thick) was done in contact holes to the Si using lift-off by putting the samples in acetone and in an ultrasonic bath for 5 min. The process was repeated with fresh acetone to ensure that all the resist (and excessive metal on top) was removed. The samples were then put in isopropanol for 2 min, rinsed in DI water, and dried with N_2_ gas.

The next step was to create a polycrystalline Zn film on the Si substrate for the NWs’ growth (Figure 1e). As an initial study, four areas of different dimensions (8 × 4, 8 × 3, 10 × 4, and 7 × 5 mm^2^) were patterned within an area of 24 × 24 mm^2^. DC sputtering was done with a 99.99% Zn target in a 99.99% Argon (Ar) gas plasma at a DC setting of 50 µA using an S150B sputter coater (HHV Ltd., West Sussex, UK) at room temperature (25°) and a working pressure of 640 Pa. A lift-off process was carried out to remove the excess Zn and to achieve a selective deposition of a Zn thin film on the patterned areas. The sputtered Zn was then annealed in air at 600 °C for 60 min to oxidize the Zn into a polycrystalline ZnO seed layer. A seed layer thickness of 100 nm was achieved with a Zn layer sputtered for 20 min. The seed layer was necessary to initiate uniform nucleation of ZnO NWs on the mirror-like Si surface and to facilitate aligned NW growth perpendicular to the substrate surface [16,29,34].

Then, ZnO NW arrays were grown on these Si substrates that were pre-deposited with a ZnO seed layer (Figure 1f). The chemical bath deposition (CBD) growth technique was employed in this process. To grow the NWs, the samples were immersed in an aqueous solution that consisted of a 1:1 ratio of 25 mmol/L of zinc nitrite (Zn (NO_3_)_2_) and 25 mmol/L of hexamethylenetetramine (C6H_12_N_4_), each of which was mixed in 250 mL of deionized (DI) water. The growth solution was kept at 90 °C for 3 h. The growth temperature was initially set at 85 °C for 5 min (to avoid overheating and ensure a stable growth temperature), and then at 90 °C for 3 h. To monitor and maintain the temperature at the set value, an external thermometer was dipped in the growth solution. Subsequently, the sample was cleaned in DI water to eliminate residuals of chemicals, dipped in acetone for 1 to 3 min to lift off the photoresist, rinsed in isopropanol, and finally dried with nitrogen gas.

In one set of samples, the ZnO NW arrays were spin-coated with SU-8 polymer (Figure 1g) at a speed of 3000 rpm for 35 s immediately after O_2_ plasma activation on the nanowires’ surfaces. This process was necessary to ensure that the SU-8 polymer adhered to the Si surface and to the NWs to provide mechanical stability and avoid short-circuiting.

During spin coating, the deposited SU-8 polymer on the densely grown NWs was left for 2 min to penetrate the NW arrays. After spin coating, the sample was placed on a hot plate (65 °C) for 1 min and then soft baked (95 °C) for 3 min. Finally, a 30/300 nm Cr/Au layer as a top electrode was deposited on a separate piece of Si using the electron beam evaporation technique.

## 3. Results and Discussion

**Figure 2:**
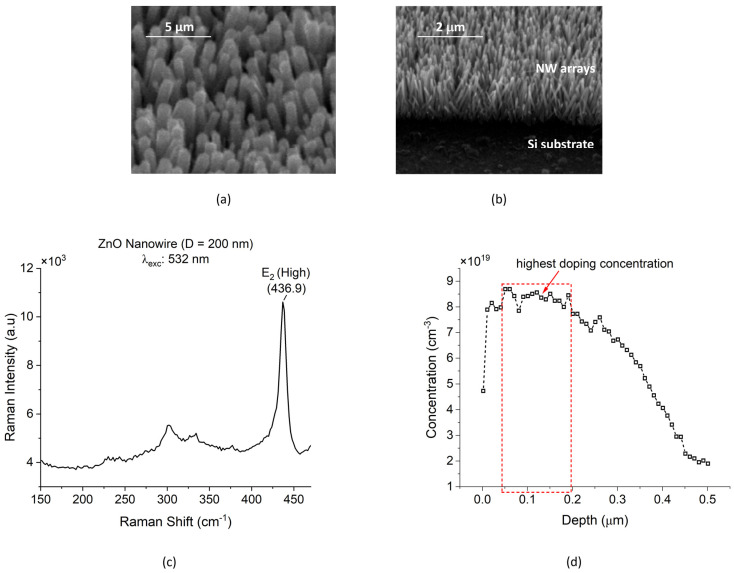
(**a**) SEM photographs of densely grown ZnO NWAs. (**b**) Cross-sectional view of area-selectively grown ZnO NWs. (**c**) Raman spectroscopy with ZnO nanowires synthesized on Si substrate by CBD. The Raman results were obtained under 532 nm wavelength, with 3 mW excitation power, and at room temperature. (**d**) ECV concentration profile of *n*-type silicon after phosphorus diffusion.

ZnO NW arrays were successfully grown in an aqueous solution using the CBD method. Four sample dice were involved in this experiment, with each die having four patterned regions. The fabrication processes and parameters were the same for each sample. The results from the scanning electron microscopy (SEM) photograph in Figure 2 show well-aligned ZnO NW arrays (NWAs) on a Si substrate. The high degree of alignment of the NWs was a result of the ZnO seed layer quality, which depends on the oxidizing/annealing temperature of the sputtered Zn [21]. ImageJ was used to determine the NW dimensions. The average NW dimensions were measured to be a diameter of *D* ~0.2 µm and a length of *L* ~1.8 µm.

The structural properties of the as-grown ZnO NWs were also observed at room temperature with a Renishaw inVia Raman Spectroscope, UK. An excitation laser wavelength of 532 nm (using a grating of 1800 l/m and a visible laser) and a laser power of 3 mW were used to measure the Raman spectra of ZnO NWs. As shown in Figure 2c, for the CVD-grown ZnO NWs, a high optical mode *E*_2_ (i.e., at a high wavenumber) was observed as the dominant peak [35]. The broad small peak around 331 cm^−1^ may be attributed to second-order Raman scattering involving acoustic phonons found with CVD ZnO NWs. The position of the *E*_2_ (high) peak at 436.9 cm^−1^ corresponded very well to the value of 437 cm^−1^ reported for both the CVD ZnO NWs and bulk ZnO, which can be taken as an indication that the NWs were free of strain. Furthermore, the linewidth of the measured *E*_2_ (high) peak was ~9.6 cm^−1^, which is close to 6 cm^−1^ reported for high-quality CVD ZnO NWs [35]. These results demonstrate the high-quality crystallinity of the CBD nanowires of the present study.

Further measurements were performed to determine the conductivity of the Si substrate on which the NWs were grown and to be used as the conductive bottom electrode of the PENGs. As already described above, the Si substrate was doped using the diffusion of phosphorus to improve the conductivity close to its surface. Before the electrochemical capacitance–voltage (ECV) measurement, a diffused reference sample was dipped in 6–7% HF for 10 min. This was necessary to remove any oxide layer. A wafer profiler CVP21 manufactured by Dage Electronics, Kaarst, Germany, was used for the ECV doping concentration profile measurement. The ECV measurements operated in two modes, periodically switching between them. This involved the use of an electrolyte solution to create a Schottky contact for the concentration measurement in the first mode. In the second mode, the sample was etched by anodic dissolution to a defined depth (e.g., 10 nm) until the next concentration measurement started using mode 1 [36,37]. The surface area of the sample that was exposed for etching was 1.13 mm^2^. The electrolyte used for the ECV measurement was 0.1 M of Ammonium bifluoride (ABF). A eutectic alloy of Ga/In was applied on the surface of the sample to create good ohmic contact with the probing pins. Figure 2d above shows the results of the ECV profile measurement of n^+^ Si. The highest concentration was measured to be in the range of (8.5 to 8.7) × 10^19^ atoms/ cm^2^ at a depth range of 50 to 190 nm.

**Figure 3:**
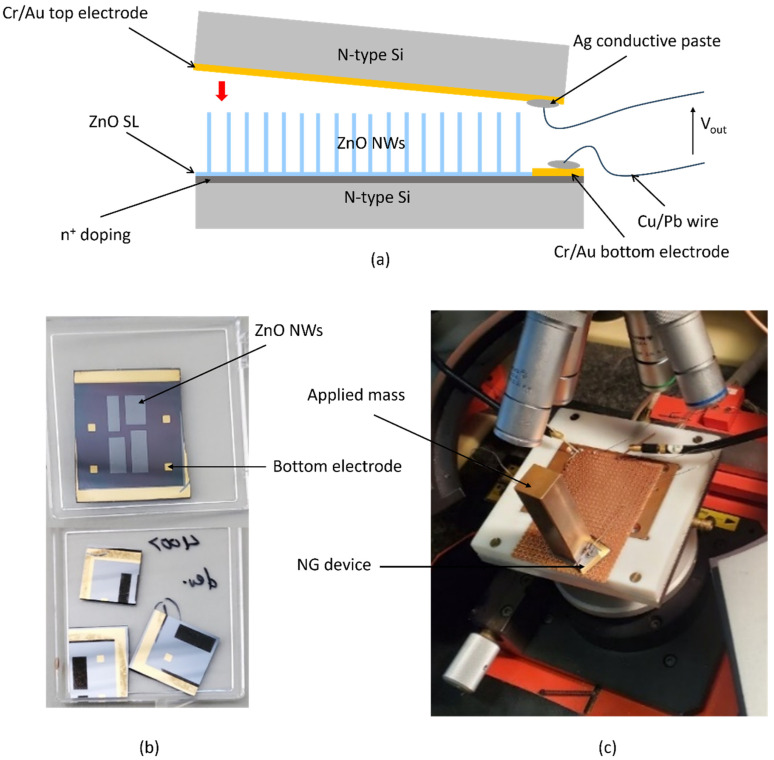
Setup and measurement of prefabricated PENG: (**a**) schematic representation of PENG device without polymer encapsulation of the NW arrays and with separate Cr/Au top electrode evaporated on bulk Si carrier die; (**b**) PENG sample and individual patterns that were cut into pieces for measurement; (**c**) PENG device mounted on a PCB and with a mass of 91.5 g positioned on the separate top electrode for impulse-type compressive force application.

Testing of the PENG device without (and with a polymer coating, not shown here) was done with a Keithley SCS4200 *I*–*V* parameter analyzer from Keithley Instruments, Inc., Cleveland, USA (input impedance: >10^13^ Ω, input leakage current: 30 pA), as shown in Figure 3.

**Figure 4:**
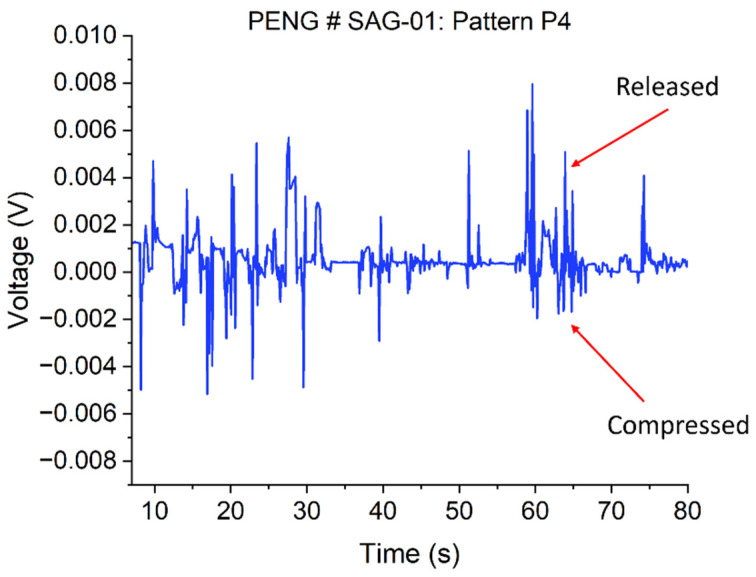
Output voltage of PENG device (NWA of 4 × 8 mm^2^ size without polymer matrix) measured with a Keithley SCS4200 *I*–*V* parameter analyzer.

Prior to testing, the four patterned areas of the sample with a 24 × 24 mm^2^ area were cut into individual dice. A bulk Si carrier die with Cr/Au deposited on it was used as a separate top electrode. The top and bottom electrodes, which formed Schottky and Ohmic contacts, respectively, were then connected to the *I*–*V* analyzer via 0.8 mm outer diameter Cu/Pb wires (Conrad Electronic SE, Berlin, Germany) connected to the electrodes’ surfaces using a conductive silver (Ag) paste. The separate top electrode was placed directly on top of the NWs (or if they were coated with a thin layer of SU-8, on top of an evaporated Au/Cr contact layer) and the output voltage was then measured via a 2-pin probe when a mass of 91.5 g was placed on it and removed from it manually and repeatedly. In this contact design, a uniform NW length was decisive to ensure electrical contact with all NWs of an array. Also, it must be noted that as the force applied to the top of the nanowires in this design was done manually, uniform compressive stress to all nanowires’ tips may not have been achieved. Again, due to the different lengths of the NWs, they may not be exposed to identical stress by the mass-loaded separate electrode on top. These effects, which will become more pronounced with large-area NWAs, are evident in Figure 4, which shows non-uniform output voltage peaks of the NG in both the compressed and released modes.

From the piezoelectric output in Figure 4 above, a maximum peak output voltage of ~7.9 mV was measured from the PENG after the release of the application of a 91.5 g mass (~0.9 N per NW, see calculation below). It can be observed that when the ZnO NWs were in contact with the top electrode and an external mass was placed on it, a negative potential was induced at the compressed side of the NWs. When the mass was released from the nanowires, a positive potential was induced. The PENG’s output voltage can be increased when the four patterns are connected in series [10], and in parallel connection, the output current can be enhanced [8,28]. When integrated on one substrate, each unit of the patterned region of the ZnO NWs can work independently.

**Figure 5:**
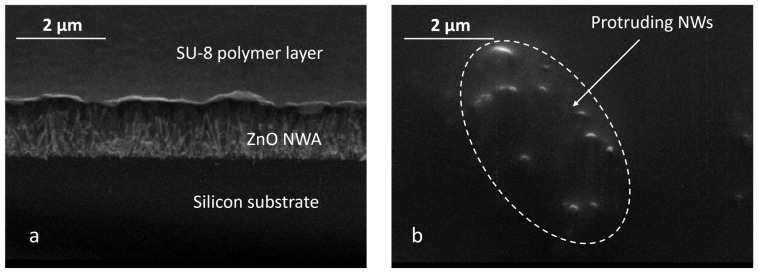
SEM images of SU-8 spin-coated on a ZnO NWA: (**a**) inclined view on the edge of a ZnO NWA with an SU-8 layer on top; (**b**) top view of the SU-8-layer spin-coated on the NWA with protruding NWs.

To study the effect of a polymer matrix, arrays of ZnO NWs with the same NW dimensions as above and the same sample size of 24 × 24 mm^2^ were spin-coated with SU-8 polymer. However, due to its high viscosity, the spin-coated SU-8 formed a thin layer of about 100 nm thick (measured with ImageJ 1.53t) on the NW array instead of embedding them, as depicted by the SEM photograph in Figure 5.

Despite the many advantages associated with SU-8 in MEMS applications, the inability of SU-8 polymer to penetrate the densely grown nanowire arrays is a major drawback. This phenomenon can be linked to the high viscosity of the SU-8 (45 cST at 25 °C) that was used in our work. Regardless of this drawback, we continued to measure the piezoelectric output of the fabricated NGs and compare the results with those from other polymers that will be utilized in our future work.

To be able to measure the piezoelectric output voltage of the fabricated NG, a separate top electrode was used as in the first experimental setup (see Figure 3a). Beforehand, a 300 nm thick Cr/Au top electrode was evaporated onto the SU-8 layer. A Si carrier die was subsequently placed on the sample and the piezoelectric output was measured. To measure the piezoelectric output, masses of 22 g, 42 g, and 91.5 g were successively put on top of the separate top electrode of the NG, generating an impulse-type compressive-mode excitation of the piezoelectric device. The height *h* from which the masses were dropped onto the NG determined its velocity when the surface was hit according to *v* = sqrt (2 × *g* × *h*) (*g* = 9.81 m/s^2^). By estimating the typical height to be *h* ≈ 0.5 cm, we obtained 0.31 m/s, which was slightly lower than the value of 0.8–0.9 m/s measured using a cam-based bending setup for flexible PENGs [38].

**Figure 6:**
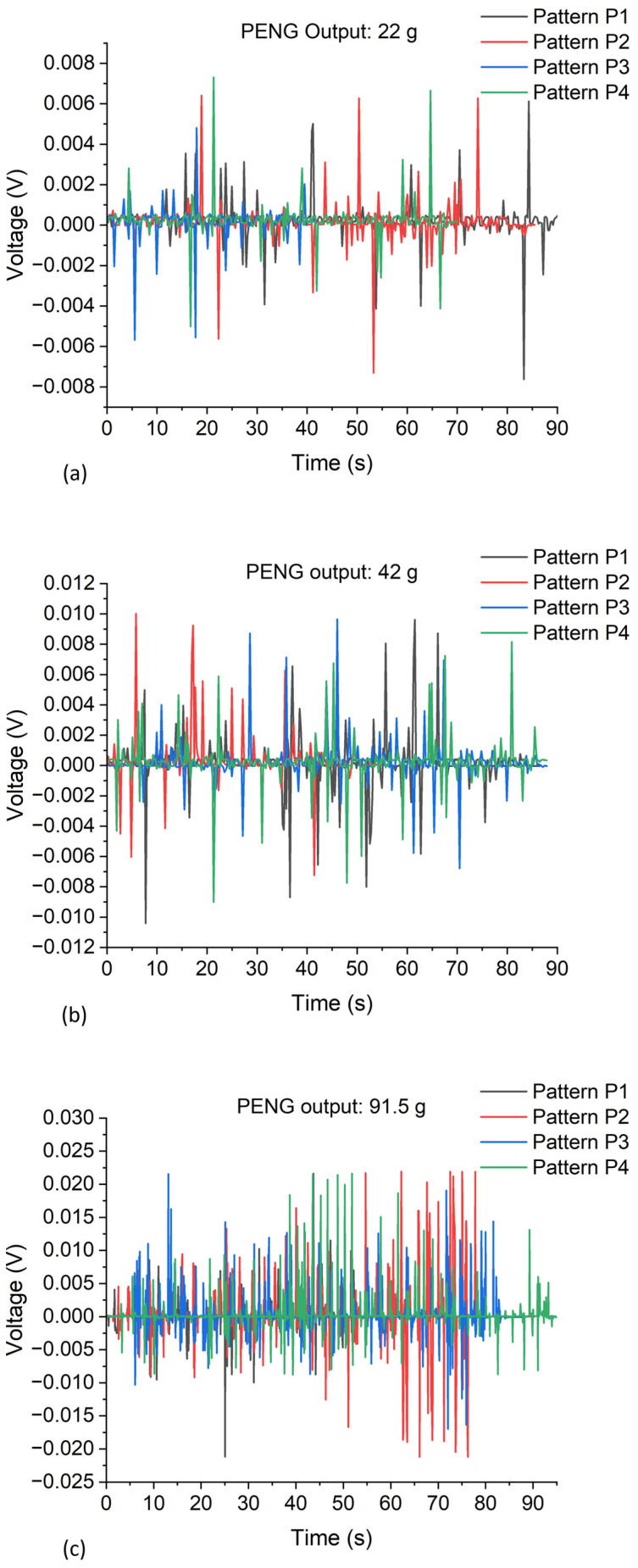
Measured output voltage due to impulse-type compressive loading for each patterned region of a PENG with an SU-8 polymer layer on top of nanowires: (**a**) results for a 22 g mass; (**b**) results for a 42 g mass; (**c**) results for a 91.5 g mass. The piezoelectric output voltage of each pattern was from the same device sample. All measurements were performed using a Keithley SCS4200 *I*–*V* parameter analyzer.

These masses were applied to each patterned region (which were of different sizes), and their output was measured using a Keithley SCS4200 *I*–*V* parameter analyzer. In the measurement process, each patterned region (P1: 4 × 10 mm^2^, P2: 5 × 7 mm^2^, P3: 3 × 8 mm^2^, P4: 4 × 8 mm^2^) of the device was measured individually using the separate top electrode. The output voltage signals from the *I*–*V* parameter analyzer for each patterned region under repeated mass loadings of 22 g, 42 g, and 91.5 g successively are shown in Figure 6. The measured peak output voltage for each pattern was observed to be almost the same, which is an indication that the nanowires’ geometry was uniformly realized by the CBD growth process that was employed. These measurements were performed to study the output behavior of the ZnO-based PENG device when the applied force (mass) was varied. The peak amplitudes of the generated voltage varied due to the not-well-defined and low-reproducibility load application and release using manually dropped mass pieces. Nevertheless, although there was some amount of noise influence on the measured signals, the piezoelectric output voltage signals were easily distinguishable.

The maximum peak output voltages from the nanogenerator device that were recorded with varied applied forces (masses) are presented in Table 1.

**Figure 7:**
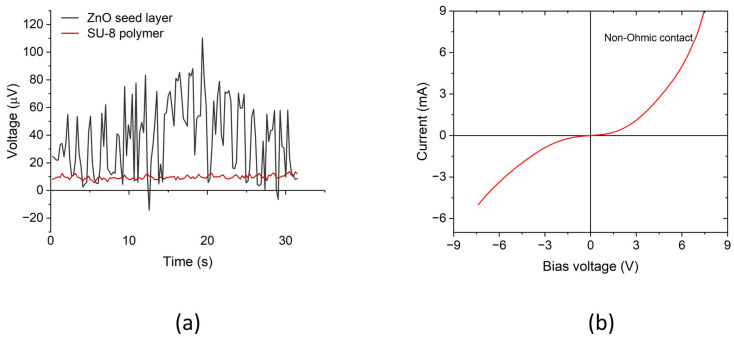
(**a**) Measured voltage from reference samples with a ZnO seed layer and a SU-8 thin layer only on Si while applying 91.5 g mass in impulse-type compressive mode. (**b**) *I*–*V* curve showing a non-Ohmic contact behavior of Au/SU-8 junction. All measurements were performed using a Keithley SCS4200 *I*–*V* parameter analyzer.

To verify that the measured voltage signal was not from environmental noise but indeed from the ZnO NWs, further measurements were performed with reference samples without ZnO NWAs. The first set of samples consisted of a ~200 nm thick ZnO seed layer (SL) deposited on a Si substrate, and the second sample was a ~2 µm thick layer of SU-8 polymer on Si. Afterward, a 30/300 nm thick Cr/Au electrode was evaporated on the ZnO SL, SU-8 polymer layer, and the back side of the Si substrates for each sample. The potential difference between the Au/Cr/ZnO SL/Si/Cr/Au and Au/Cr/SU-8/Si/Cr/Au layers was measured using the *I*–*V* parameter analyzer when a mass of 91.5 g was manually applied in the repeated-compression mode. The measurement procedure was the same as with the PENGs presented earlier. The results from both measurements are presented in Figure 7a, which shows the maximum peak output voltage of ~110.3 µV for the ZnO SL sample and ~13.7 µV for the SU-8 sample. It can also be seen that there was no clear piezoelectric behavior of the measured signals, as the signals exhibited almost no negative output and mainly positive signals upon both the compression and release of the applied mass. We can therefore conclude that the output signals from our nanogenerator device were actually piezoelectric output voltages generated in the ZnO NWAs.

Again, to verify the formation of a Schottky contact on the ZnO nanowires, an *I*–*V* curve of the NG was obtained using the *I*–*V* parameter analyzer. To do this, the current through the NG was measured as the voltage was increased from −8 V to +8 V. The resultant curve in Figure 7a depicts a Schottky-like, non-Ohmic contact behavior of the device, which was assigned to the Au/Cr/SU-8/ZnO junction/interface. In a further measurement, we found an Ohmic characteristic for the ZnO/*n*^+^Si/Cr/Au bottom contact [39].

To estimate the performance and better understand the electric potential development in the ZnO-NW-based NG device, a single ZnO NW under a compressive force was simulated using COMSOL Multiphysics. The axial compressive force was applied along the *z*-axis of the NW with a <100> crystal orientation, i.e., Zn surface polarity. During the simulation, the following boundary conditions were defined: (i) the boundary load, i.e., the face where the compressive force was applied, was defined as the entire top face of the NW; (ii) the fixed constraint, which modeled the structurally blocked face of the system with respect to the *x*, *y*, and *z* displacements, was defined as the bottom face of the NW; and (iii) the ground, or zero electric potential plane, was also defined as the bottom face. Points (i) and (ii) were mechanical conditions and point (iii) represented an electrical condition [12,40]. In this simulation, it was assumed that the whole nanowire top surface was in contact with the applied force. We would like to emphasize here that due to the complexity of implementing a doping profile in COMSOL coupled with its simulation challenges, the built-in silicon material in the COMSOL material library was utilized instead of the phosphorus-doped contact layer on *n*-type silicon. The material properties of silicon, ZnO, and SU-8 that were used in our simulation are presented in Table 2, Table 3 and Table 4, respectively.

When the ZnO NW was subjected to an axial compressive force of 0.9 nN, for a single NW of length *L* = 1800 nm and diameter *D* = 200 nm, a generated potential of −6.5 mV was calculated at the top, with the zero electrical potential plane defined at the bottom of the NW as a boundary condition for the FEM. A force of 0.9 nN was applied to the single NW in accordance with the experimentally applied force (91.5 g corresponding to a gravitational force of ~0.9 N) to an array of ~1 × 10^9^ NWs (estimated from the base area of a single NW of ~3 × 10^−8^ mm^2^, which was assumed to be arranged in a closed-packed array in the device area of 4 × 8 mm^2^ = 32 mm^2^). The resulting areal density of ~3 × 10^9^ NWs/cm^2^ compared well with the experimental result [21]. Nevertheless, the small deviation of the simulated potential of −6.5 mV from the measured maximum peak voltage of −7.9 mV could be attributed to a too-rough estimation of the number of NWs in the array considering the non-ideal NW arrangement visible in Figure 2. A non-uniform height distribution may lead to the application of a larger force than 0.9 nN to the NWs of a larger height. Furthermore, NW bending will have to be considered in addition to compression in a more realistic simulation due to the non-perfect vertical alignment of the ZnO NWs. In the case of coexisting piezoelectric phases with Zn and O surface polarities (i.e., if not all NWs have a Zn surface polarity as assumed in FEM), the induced negative and positive potentials, respectively, could have canceled out. Finally, the screening of the piezoelectric field by free electrons in the ZnO NWs was not considered in our FEM, i.e., doping, surface traps, etc., were not considered in our COMSOL simulations. Therefore, the presented COMSOL simulation results only give an estimate of the output voltage values, which, nevertheless, can be used to evaluate the magnitude of experimental results.

**Figure 8:**
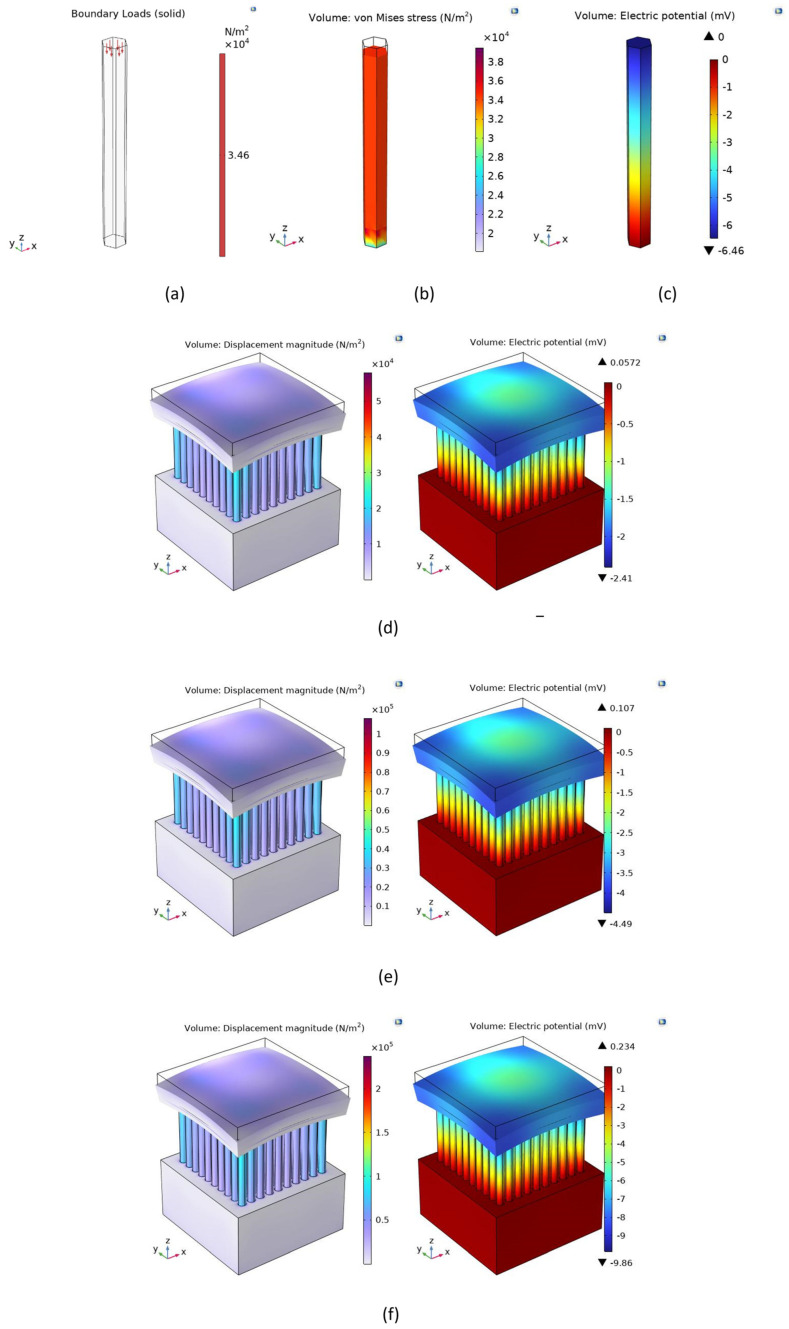
FEM modeling of a single ZnO NW and an NWA with SU-8 layer on top: (**a**) boundary load indicating direction of applied force of 0.9 nN to single nanowire; (**b**) axial displacement; (**c**) potential distribution of the ZnO NW; (**d**–**f**) axial displacement and potential distribution of a ZnO NW array of 3 × 3 µm^2^ area with SU-8 layer on top and under axial compressive force of 22 nN, 41 nN, and 90 nN, respectively. NW dimensions correspond to average values measured using SEM and ImageJ.

For an evaluation of the measured performance of the NG devices based on ZnO NWAs with SU-8 top layer, an array area of 3 µm × 3 µm containing 10 × 10 NWs was simulated using COMSOL Multiphysics under an axial and static compressive force. Distributed forces of 22 nN, 41 nN, and 90 nN were applied by inducing compressive stresses of 2.444 kPa, 6.625 kPa, and 10 kPa to the NWA area along the *z*-axis of the NWAs. A 100 nm thick SU-8 polymer layer was added on top of the NWA followed by a 30/300 nm Cr/Au layer as the top electrode. The calculations from the simulations gave generated piezoelectric potentials of −2.4 mV, −4.5 mV, and −9.9 mV, respectively, which were much smaller than the maximum measured values of the four NWA patterns but nearly agreed with their averages. The results from the simulation with applied forces of 0.9 nN for a single NW, as well as 22 nN, 42 nN, and 90 nN to an NWA of 100 NWs in a 3 × 3 µm^2^ area, are depicted in Figure 8 below.

To mitigate the adverse effect of manually applying a force, measurements were repeatedly done for improved statistics. Mean values of measured peak output voltages and their corresponding standard deviations, which were realized from the experimental and simulation results in this work, are summarized in Table 5 below. For this, the peak values of both the polarity of the NG output measured upon both load application and release were read from Figure 6a–c and averaged. Standard deviations were calculated and taken as the measurement uncertainty [38].

From Table 5, when the applied mass was increased in steps of 22 g, 42 g, and 91.5 g, there was a corresponding output voltage increment for each patterned region of the PENG device. The trend was the same for the simulation results when the applied force was increased in steps of 22 nN, 41 nN, and 90 nN, which was to be expected.

**Figure 9:**
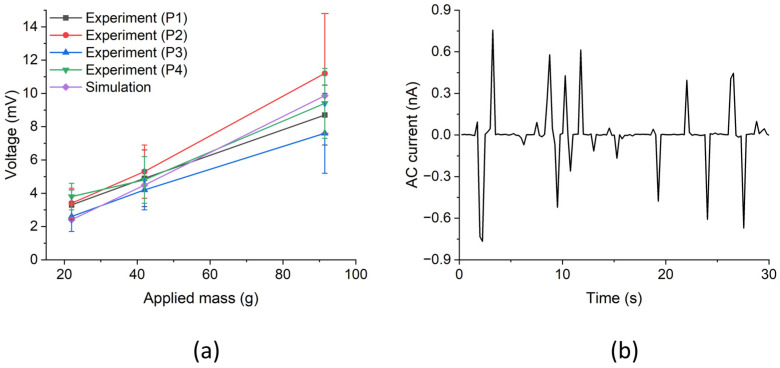
(**a**) Experimental and simulation results with NWAs of the patterned regions P1 to P4 of the PENG device. The experimental loading masses were 22 g, 42 g, and 91.5 g, which corresponded to applied simulation forces of 22 nN, 41 nN, and 90 nN on a 3 × 3 µm^2^ array area, respectively. (**b**) Output current measured with the NWA in P1 at repeated application/release of a load of 91.5 g using the *I*–*V* parameter analyzer.

The large uncertainties of the measured data could be attributed to the reproducibility issues of the used manual force application to the nanowire arrays, i.e., the number of nanowires in contact with the applied force may have varied. Nevertheless, within these error bounds, as is visible in Figure 9, linear dependences of the average output voltage on the applied mass can be observed for all array areas, as expected by the results of the FEM.

The peak values of open-circuit voltage *V*_oc-peak_ and short-circuit current *I*_sc-peak_ can be used to calculate the peak power *P*_peak_ = *V*_oc-peak_ × *I*_sc-peak_ of a PENG [38]. To characterize our nanogenerator device in terms of the power output, it was connected to a resistive load of 100 kΩ, which was much lower than the input impedance of the Keithley analyzer (10 TΩ). From a measurement with the NWA in pattern P1 with repeated applications/releases of a load of 91.5 g, we obtained the current output shown in Figure 9b. Here, the peak current amplitudes were much higher than the leakage current of the Keithley analyzer of <30 pA. Taking the peak value of 0.8 nA as an estimate for *I*_sc-peak_ and the corresponding *V*_oc-peak_ = 21.6 mV (Table 1), we calculated a peak output power of ~0.017 nW at a load of 0.9 N. The active volume, where the piezoelectric power was generated, was given by the considered NW array area times the sum of NW and polymer heights [38]. With the corresponding values of 0.4 cm^2^ × 2 µm, the power density per active volume amounted to 0.22 µW/cm^3^.

The presented results are consistent with the literature and may be a basis for optimizing the NG output in the future. In a similar work, C. Oshman et al. [12] reported a PENG based on a ZnO NW array that was spin-coated by PDMS and contacted by sputtered Ti/Au, yielding an (open-circuit) output voltage of 3 mV under an applied compressive load of 0.451 N. This compares very well with the range of 2.6 ± 0.9 mV to 3.8 ± 0.8 mV we found with our devices at a load of 22 g (corresponding to a gravitational force of 0.22 N; see Table 5). Furthermore, the calculated peak power density per active volume of 0.22 µW/cm^3^ from our device is very similar to the value ~288 nW/cm^3^ reported by C. Opoku et al. [41] with a ZnO NW array coated by a PDMS polymer. Nevertheless, much larger values of open-circuit voltage (272 mV) and peak power (17 nW) under 3 N compressive force will be possible, which is related to the seed layer quality and load resistor adjustment [42].

The piezoelectric coefficient d_33_ of ZnO nanowires, which is the parameter that associates polarization with stress at a constant field and strain with the electric field at constant stress was calculated in our experiment. The bulk sample piezoelectric d_33_ measurements were carried out using the new measurement tool ESPY33 developed under project Nanowires (EMPIR 19ENG05), which enables accurate and traceable measurement of a charge that develops across a piezoelectric sample undergoing a compressive cyclic stress [43]. The ESPY33 applies a known cyclic mechanical force to the sample (device under test) between two aligned mechanical probes. A DC static preload can be applied to hold the sample in place whilst the AC force is executed. The charge is measured using electrical contact probes that also act as mechanical probes. Details of the device are found on the company website [43] and in patent GB2572334A [44]. Charge and force are recorded using digital electronics and storage scope and the effective piezoelectric charge coefficient is calculated using *d*_33_ = (*Q*/*A*_1_)/(*F*/*A*_2_), where *Q* is the charge in C; *F* is the force in N; and *A*_1_ and *A*_2_ are the electrode areas and force areas, which, in the cases discussed in this paper, were equivalent. Details of this measurement method are found in a metrology handbook [45].

The sample was prepared using a silicon bottom wafer (1–10 Ωcm, thickness 275 µm) and four lithographically patterned NWA areas (NWs of 200 nm diameter, 1.8 µm in length) with vias providing top access to the bottom planar electrode. This sample was modified such that the earth (bottom electrode) via pads were connected to the ESPY33 tool’s bottom probe. The sample had four top patterns processed at various locations and a thin SU-8 polymer top infill that allowed Cr/Au top electrodes to be deposited. The device was loaded into the ESPY33 tool such that the direction of the load was along the long axes of the nanowires. The ESPY33 tool’s top probe contacted the top surface of the sample (the pattern or metalized surface) and the bottom probe contacted the backside of the silicon wafer that was electrically connected to the device’s bottom electrode.

The ESPY33 tool was previously calibrated against a standard mass force and known reference capacitor and voltage source, which enabled the forces and charges to be traceably measured. The force was measured using an instrumented load cell and charge via a transimpedance virtual earth operational amplifier. The tool was then used to measure the piezoelectric charge response of two standard reference artifacts: quartz and lithium niobate. The values recorded were within 1% of the true values for these single-crystal piezoelectric materials (quartz: d_11_ = 2.27 ± 0.30 pC/N, lithium niobate = 20.0 ± 0.2 pC/N).

**Figure 10:**
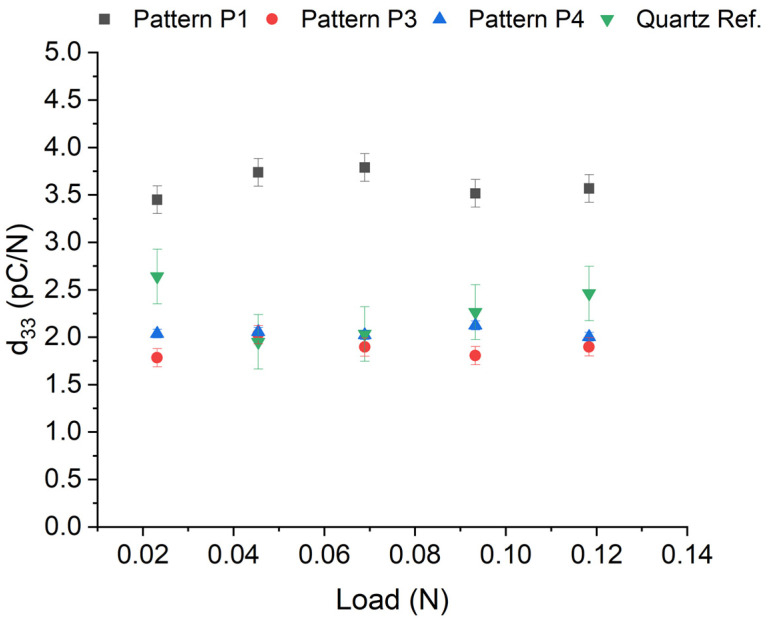
Effective bulk piezoelectric charge coefficients measured using the ESPY33 tool.

Identical pre-loads of 0.1 N were applied to the samples and a small signal force of 100 mN was applied in increments of 10 mN to examine any load dependence of the piezo response. The results for the ZnO samples are shown in Figure 10. All four patterns of the sample were examined. The theoretical value of effective ‘*d*_11_ (or equivalent *d*_33_)’ response along the nanowire length from the compressive force is of the order of 9.9 pC/N. Our data indicate that an effective non-zero and statistically significant ‘bulk’ piezoelectric response was measured in the sample, with nominal values of 1.9, 2.1, and 3.6 ± 0.5 pC/N for three out of the four working patterns (P1, P3, and P4).

Values of up to 3.6 ± 0.5 pC/N were found with the patterned NWAs, which had a thin (~100 nm) SU-8 top layer. Here, the larger diameter of ~200 nm and the semiconducting properties of the NWs may have determined their piezoelectric performance. This interpretation is supported by piezoresponse force microscopy (PFM) with single ZnO NWs, where the effective piezoelectric coefficient *d*_33_^eff^ was observed to decrease with the NW diameter and SL thickness [46]. For a diameter and SL thickness of ~210 nm and 150 nm, respectively, which are close to those of the present study (200 nm and 100 nm, respectively), *d*_33_^eff^ was between 2.9 pm/V and 3.8 pm/V, i.e., in reasonable agreement with our findings.

## 4. Conclusions

A novel chemical bath deposition (CBD)-based two-step ZnO nanowire (NW) process was described and grew well-aligned ZnO NW arrays (NWAs) area-selectively on Si, beginning with a DC-sputtered Zn precursor layer, which was then annealed in air to form a ZnO SL. An advantage of this growth technique is that the ZnO seed layer can be patterned by lift-off of the Zn precursor layer using a photoresist.

The geometry of the NWs was characterized by scanning electron microscopy (SEM) to determine their length *L* and diameter *D*, yielding average values of *L* ~1.8 µm and *D* ~0.2 µm. The SEM images also revealed that high-density NWAs of vertically aligned orientation were grown from the ZnO seed layer. This is an indication that seed layers by Zn sputtering followed by oxidation were suitable for growing hexagonal-top-face NWs with good alignment normal to the substrate surface plane. This method enabled area-selective growth of separated patterns of ZnO NWAs, which could be connected in series to provide piezoelectric nanogenerators (PENGs), with elevated output voltages as needed for powering standalone devices.

Based on this process, PENGs were fabricated with and without a polymer layer on top of the NW array. A separate top electrode with Cr/Au evaporated on a Si carrier was placed on the single NWA patterns. We found peak output voltages of ~7.9 mV upon releasing the loading of bare ZnO NW arrays. Further testing of the PENG device was done with varied masses of 22 g, 42 g, and 91.5 g on further NWA patterns, in this case with an SU-8 top layer. For comparison, the piezoelectric response of a bare single ZnO NW and a 3 × 3 µm^2^ NWA pattern containing 100 NWs with a SU-8 polymer layer on top were modeled using COMSOL Multiphysics under a compressive force of 0.9 nN for the single NW and 22 nN, 41 nN, and 90 nN for the 3 × 3 µm NWA. Good agreement was found in all cases between the simulation and average measured values. According to expectation, the dependence between the output voltage and applied load was observed to be nearly linear. Maximum peak values of 21.6 mV and ~0.017 nW at a load of 0.9 N were found for the open-circuit voltage and the output power, respectively.

The next steps will aim at an improvement of the output potential of patterned ZnO NW arrays by optimizing the seed layer quality for better morphology of the ZnO NWs. and the embedding process using alternative polymer materials. A micro-shaker testing platform will be utilized for different cases of dynamic excitation (periodic, impact, noise).

## Figures and Tables

**Table 1:** Experimental results of patterned ZnO-based PENG devices at varied applied force (mass). Experimental output potential values are measured maximum peak voltage values.

Patterned Region	Array Area (mm^2^)	Top Polymer	Maximum Peak Output Potential (mV)		
			*F* = 22 nN (22 g)	*F* = 41 nN (42 g)	*F* = 90 nN (91.5 g)
P1	4 × 10	100 nm SU-8 layer	−7.6	−10.4	21.6
P2	5 × 7		−7.3	10.0	21.9
P3	3 × 8		−5.7	9.6	21.6
P4	4 × 8		−7.3	9.0	21.6

**Table 2:** Material properties of silicon from COMSOL material library.

Property	Expression	Unit
Relative permeability	1	1
Electrical conductivity	1 × 10^−12^	S/m
Coefficient of thermal expansion	2.6 × 10^−6^	1/K
Heat capacity at constant pressure	700	J/(kg × K)
Relative permittivity	11.7	1
Density	2329	kg/m^3^
Thermal conductivity	130	W/(m × K)
Young’s modulus	170 × 109	Pa
Poisson’s ratio	0.28	1
Refractive index, real part	3.48	1
Refractive index, imaginary part	0	1
Relative permeability	1	1

**Table 3:** Piezoelectric properties of ZnO from COMSOL material library.

Property	Value	Unit
Density	5680 kg/m^3^	kg/m^3^
Relative permittivity	$\left[\begin{array}{ccc} 8.5446 & 0 & 0\\ 0 & 8.5446 & 0\\ 0 & 0 & 10.204 \end{array}\right]$	1
Elasticity matrix, Voigt notation	$\left[\begin{array}{cccccc} 2.09714\times 10^{11} & 1.2114\times 10^{11} & 1.05359\times 10^{11} & 0 & 0 & 0\\ 1.2114\times 10^{11} & 2.09714\times 10^{11} & 1.05359\times 10^{11} & 0 & 0 & 0\\ 1.05359\times 10^{11} & 1.05359\times 10^{11} & 2.11194\times 10^{11} & 0 & 0 & 0\\ 0 & 0 & 0 & 4.23729\times 10^{11} & 0 & 0\\ 0 & 0 & 0 & 0 & 4.23729\times 10^{11} & 0\\ 0 & 0 & 0 & 0 & 0 & 4.42478\times 10^{11} \end{array}\right]$	Pa
Coupling matrix, Voigt notation	$\left[\begin{array}{cccccc} 0 & 0 & 0 & 0 & -0.480508 & 0\\ 0 & 0 & 0 & -0.480508 & 0 & 0\\ -0.567005 & -0.567005 & 1.32044 & 0 & 0 & 0 \end{array}\right]$	C/m^2^

**Table 4:** Material properties of SU-8 from COMSOL material library.

Property	SU-8 IN	SU-8 OUT	Unit
Density	1217.8	1217.8	kg/m^3^
Relative permittivity	1	1	1
Coefficient of thermal expansion	8.71× 10^−5^	2.78 × 10^−4^	1/K
Shear modulus	1.2 × 10^9^	1.4 × 10^8^	N/m^2^
Young’s modulus	3 × 10^9^	4 × 10^9^	Pa
Poisson’s ratio	0.33	0.29	1
Tangent coefficient of thermal expansion	8.71 × 10^−5^	2.78 × 10^−4^	1/K
Thermal strain	−5.8 × 10^−5^	−1.78 × 10^−4^	1

**Table 5:** Mean values of experimental and simulation results of the open-circuit output voltage of patterned ZnO-based PENG devices at varied applied forces. The nanowires had an SU-8 polymer layer on top. The experimentally applied masses of 22 g, 42 g, and 91.5 g corresponded to simulation of compressively applied forces of 22 nN, 41 nN, and 90 nN on a (3 × 3 µm^2^ array area containing 100 NWs, respectively. Diameter (~200 nm), height (~1.8 µm), and density (~3 × 10^9^ NWs/cm^2^) of the NWs used for simulation correspond to experimentally determined values.

Patterned Region	Array Area (mm^2^)	Top Polymer	PENG Output Potential at Varied Applied Force					
			*F* = 22 nN (22 g)		*F* = 41 nN (42 g)		*F* = 90 nN (91.5 g)	
			Measured Value (mV)	Simulated Value (mV)	Measured Value (mV)	Simulated Value (mV)	Measured Value (mV)	Simulated Value (mV)
P1	4 × 10	100 nm SU-8 layer	3.3 ± 0.9	−2.4	4.9 ± 1.7	−4.5	8.7 ± 1.8	−9.9
P2	5 × 7		3.4 ± 0.9	−2.4	5.3 ± 1.6	−4.5	11.2 ± 3.6	−9.9
P3	3 × 8		2.6 ± 0.9	−2.4	4.2 ± 1.2	−4.5	7.6 ± 2.4	−9.9
P4	4 × 8		3.8 ± 0.8	−2.4	4.8 ± 1.4	−4.5	9.4 ± 2.1	−9.9

## Data Availability

The data presented in this study are available on request from the corresponding author.
